# Real time surveillance of COVID-19 space and time clusters during the summer 2020 in Spain

**DOI:** 10.1186/s12889-021-10961-z

**Published:** 2021-05-21

**Authors:** Nicols Rosillo, Javier Del-guila-Meja, Ayeln Rojas-Benedicto, Mara Guerrero-Vadillo, Marina Peuelas, Clara Mazagatos, Jordi Seg-Tell, Rebeca Ramis, Diana Gmez-Barroso

**Affiliations:** 1 Servicio de Medicina Preventiva. Centro de Actividades Ambulatorias, 6 planta, Bloque C, Hospital Universitario 12 de Octubre. Avenida de Crdoba, s/n, 28041 Madrid, Spain; 2 Centro Nacional de Epidemiologa, Instituto de Salud Carlos IIII, Calle de Melchor Fernndez Almagro 5, 28029 Madrid, Spain; 3 Servicio de Medicina Preventiva, Hospital Universitario de Mstoles, Calle Ro Jcar, s/n, 28935 Mstoles, Spain; 4 Consorcio de Investigacin Biomdica en Red de Epidemiologa y Salud Pblica (CIBERESP), Instituto de Salud Carlos III, Calle Monforte de Lemos 3-5, 28029 Madrid, Spain

**Keywords:** COVID-19, Spatial analysis, Clusters, Spain, Surveillance

## Abstract

**Background:**

On June 21st de-escalation measures and state-of-alarm ended in Spain after the COVID-19 first wave. New surveillance and control strategy was set up to detect emerging outbreaks.

**Aim:**

To detect and describe the evolution of COVID-19 clusters and cases during the 2020 summer in Spain.

**Methods:**

A near-real time surveillance system to detect active clusters of COVID-19 was developed based on Kulldorfs prospective space-time scan statistic (STSS) to detect daily emerging active clusters.

**Results:**

Analyses were performed daily during the summer 2020 (June 21st August 31st) in Spain, showing an increase of active clusters and municipalities affected. Spread happened in the study period from a few, low-cases, regional-located clusters in June to a nationwide distribution of bigger clusters encompassing a higher average number of municipalities and total cases by end-August.

**Conclusion:**

STSS-based surveillance of COVID-19 can be of utility in a low-incidence scenario to help tackle emerging outbreaks that could potentially drive a widespread transmission. If that happens, spatial trends and disease distribution can be followed with this method. Finally, cluster aggregation in space and time, as observed in our results, could suggest the occurrence of community transmission.

## Background

The World Health Organization declared COVID-19 a Public Health Emergency of International Concern (PHEIC) on January 30th [1] and a global pandemic on March 11th, due to the increase in disease cases and its rapid spread throughout the world [2].

On March 14th, the Spanish government declared a state of alarm, a constitutional prerogative that allows the temporary suspension of movements [3], and established a confinement of virtually the entire population (except essential workers), which played an important role in the control of the first pandemic wave. The state of alarm ceased on June 21st [4], after a period of progressive de-escalation of COVID-19 control measures. From there on, the epidemiological situation significantly changed with daily new cases greatly reduced and testing capacity improved. Following de-escalation, new challenges arose and the focus shifted from curve bending to outbreak and transmission chain control under the Control and Surveillance Strategy in the transition phase of the COVID-19 pandemic implemented by the Ministry of Health of Spain [5].

With the lifting of measures, small outbreaks began to occur, at first related to vulnerable collectives, such as workers from agriculture or meat-processing factories as happened in other parts of Europe, related to poor living conditions, low socioeconomic status and marginalized collectives [6]. These outbreaks progressively grew in cases and locations affected and, eventually, community transmission was suspected. By August, the number of daily new cases had greatly increased [7], and new control measures were reevaluated, ranging from limiting social gatherings up to small-contained lockdowns.

Space-time Scan Statistic (STSS) has been widely used since its first development by Kulldorf [8]. Originally designed for retrospective analysis of chronic conditions and mortality [9], a prospective version was also proposed [10] for surveillance data [11], which was quickly aimed toward outbreaks detection [12], like dengue fever [13] and malaria [14]. STSS analysis has also been applied to monitor the emergence of new active clusters of COVID-19 in the USA [15, 16] or Bangladesh [17].

Our aim is to detect and describe the evolution of COVID-19 clusters and cases during the 2020 summer following the end of the State of Alarm (21 June 31 August) in Spain using a real-time scan statistic prospective analysis, and to assess its implementation as a tool for daily epidemic surveillance.

## Methods

### Administrative distribution of Spain

**Map 1:**
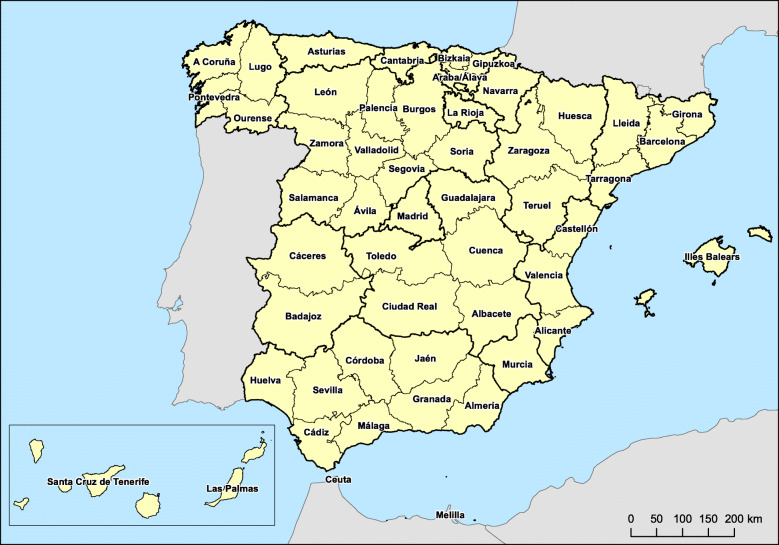
Political map of Spain

Spain is composed of 17 Autonomous Regions and 2 Autonomous Cities. These are subdivided into 52 provinces (shown in Map1). The smallest territorial administrations are municipalities. As of January 1, 2020, there are 8131 of them. Their number and extension are heterogeneous among territories. We used 2019 population data for each Spanish municipality from INEbase, the National Statistics Institute of Spain official database [18].

### Data collection and inclusion criteria

COVID-19 cases, recorded by Autonomous Regions and Autonomous Cities as part of the National Epidemiological Surveillance Network (RENAVE in Spanish), are stored in the Spanish Surveillance System electronic platform (SiViES in Spanish), and managed by the National Centre for Epidemiology. This database contains information on demographic, epidemiological, clinical and laboratory aspects. The official protocols implemented during this period aimed at the early detection of symptomatic cases and, if possible, the active search for cases (both symptomatic and asymptomatic) among the contacts of the cases [5]. A COVID-19 case is considered confirmed, and therefore is notified to RENAVE if there is a positive polymerase chain reaction (PCR) test, or an ELISA-based serological test (IgM) in patients with suitable symptoms and negative PCR [5].

For the analysis, the date assigned to each case was computed as the imputation date. The date of symptom onset and the date of diagnosis were used for symptomatic and asymptomatic cases, respectively. When date of symptom onset was missing, the imputation date was calculated as diagnosis date minus 3 days (median time from symptom to diagnosis in our study) [5]. Cases with none of these dates available were excluded.

### Prospective Poisson space-time scan statistics

A prospective version of Poisson STSS was employed to detect active clusters at municipal level in Spain. STSS is characterized by a cylindrical window where the base is the spatial scanning window and the height corresponds to time. The method scans through space and time analyzing each possible geographic area and time range. Thus, we obtain an extensive number of cylinders for the entire area of analysis.

As we assume the number of COVID-19 cases follow a Poisson distribution, under the null hypothesis (Ho) the risk within the cylinder is constant while under the alternative hypothesis (H_1_) the risk inside the cylinder differs from outside. As stated by Kulldorf [10] and Desjardins [15], the equation below (Eq. 1) is used to calculate, under the null hypothesis, the expected number of cases (**) where *p* is the population in cylinder base area, *C* the number of COVID-19 cases in Spain and *P* the total Spanish population.

$$ \mu =\rho \ast \frac{C}{P} $$

Then, a likelihood ratio test (Eq. 2) [8, 15] for each cylinder is calculated taking into account the number of cases and the population at risk, the higher the value the less likely that the detected cluster occurred by chance.

$$ \frac{L(Z)}{L_0}=\frac{{\left(\frac{n_z}{\mu (Z)}\right)}^{n_z}{\left(\frac{N-{n}_z}{N-{\mu}_z}\right)}^{N-{n}_z}}{{\left(\frac{N}{\mu (T)}\right)}^N} $$

The ratio is defined by the quotient between the likelihood of the cylinder Z, *L(Z)*, and, *L*_*0*_, the likelihood under the null hypothesis. The variables are: n_z_, the number of COVID-19 cases within the cylinder area; *(Z)*, the expected cases calculated by the Eq. 1; *N*, the total number of cases in Spain across the time range and *(T)* the expected cases in the area across the time range.

The significance was evaluated for each detected cluster using the Monte Carlo test. The obtained likelihood ratio was compared to likelihood distribution calculated with the permutations of the data. For each analysis (day), clusters are presented in order of likelihood ratio of occurrence.

To easily compare the relative risk between the detected clusters, a relative risk for each cluster is calculated with the formula below (Eq. 3) [13, 15]:

$$ RR=\frac{c/e}{\left(C-c\right)/\left(C-e\right)} $$

where *c* and *e* are the observed and expected cases in a cluster, respectively, and *C* is the total number of cases in Spain.

### Analysis strategy and data presentation

SiViES data was retrieved during September 2020, and a daily prospective analysis was emulated for the study period. To this end, only cases recorded up to the analyzed day were taken in consideration, and only active clusters were reported, not accounting for outcomes of following days. Table1 summarizes parameters used in the STSS analysis. The cylindrical window maximum radius for our analysis was 25km (mean distance between municipalities in Spain) whereas the maximum time period of aggregated analysis was from 2 to 7days (to include median incubation period of 5days).

**Table 1:** Parameters used for the Prospective STSS analysis

Probability Model	Discrete Poisson
**Spatial window shape**	Circular
**Maximum Spatial window area**	25km radius
**Minimum Temporal cluster duration**	2days
**Maximum Temporal cluster duration**	7days
**Maximum Monte Carlo permutations**	999
**P-value significance**	p-value <0.005

Daily number of active clusters is calculated. A detailed review of two specific days will be described (June 25th and August 1st) like an example, analyzing the following individual cluster information: time period, locations included, cluster population, cluster radius, cluster *p*-value, observed cases, expected cases and relative risk. Lastly, mean cluster radius, mean number of locations included, and mean cluster duration in days through the study period are graphically represented.

### Software

STSS was performed using the SaTScan v9.6 software (https://www.satscan.org/). The automatization of the process was carried out with R Software 4.0.2 and the package rsatscan [19]. Additional graphics and maps were obtained with ggplot2 [20] and ArcMap10.3. A dynamic online viewer was developed using the packages Leaflet [21] and shiny [22] and uploaded to a server to be consulted by the public.

## Results

### Epidemic curve and cluster evolution during 2020 summer

**Fig. 1:**
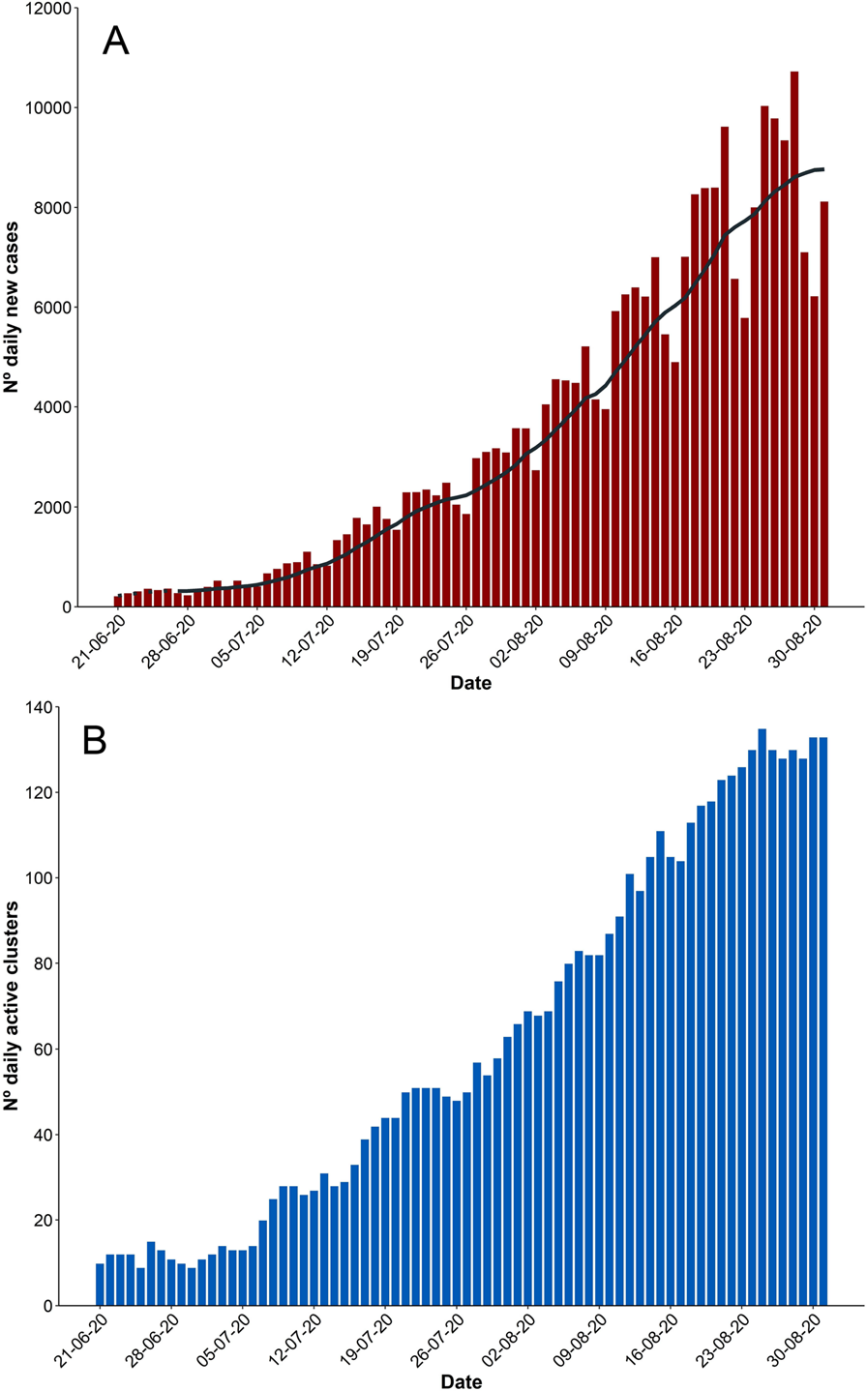
Epidemic and daily cluster curves, June 21st August 31st 2020. Note: **a** Epidemic curve of COVID-19 daily new cases as reported to the National Surveillance System (RENAVE). The black line represents the 7-day moving average of daily cases. **b** Daily active clusters detected after running STSS analysis for each day, cases reported from the National Surveillance System

From June 21st to August 31st, a total of 257,881 COVID-19 cases were registered through RENAVE. Figure1a shows the epidemic curve, where an increasing trend of daily new cases can be appreciated beginning in July, shortly after the full lifting of control measures.

Cluster evolution followed a similar trend (Fig.1b). Its spatio-temporal evolution can be consulted in a Shiny web application, available online at the following link: (https://coviddifusion.isciii.es/SpSumClus/). As can be observed in the viewer, at the first day of our study, June 21th, a total of 7 active clusters were active in Spain, mostly located in the north-eastern regions of Aragn, Catalua and Pas Vasco, which encompass a geographical region called Ebros valley. Since then, and under our analysis parameters, a constant increase in the number of clusters was noted, reaching around 50 active clusters by late-July, and 100 by the second week of August. It must be noted that the orange-colored cluster represents the most likely in terms of likelihood.

In July, these clusters were grouped into two areas: Ebros valley and Madrid. The Mediterranean coast was also affected, reaching Andaluca. As the time passed, cluster distribution became more homogeneous throughout the national territory. In August, clusters had extended from Madrid to neighboring areas of Castilla-La Mancha and Castilla y Len. By August 31st, cluster number had grown to 129, extending over almost every region of Spain with notable exceptions such as Asturias.

The location of the most likely cluster has changed throughout the study period. Initially, it was placed in some municipalities of Huesca and Lleida, and later, around the city of Zaragoza. Later on, it was displaced to the Barcelona metropolitan area by July 20th, and from August 8th to the surroundings of the capital city of Madrid.

**Map 2:**
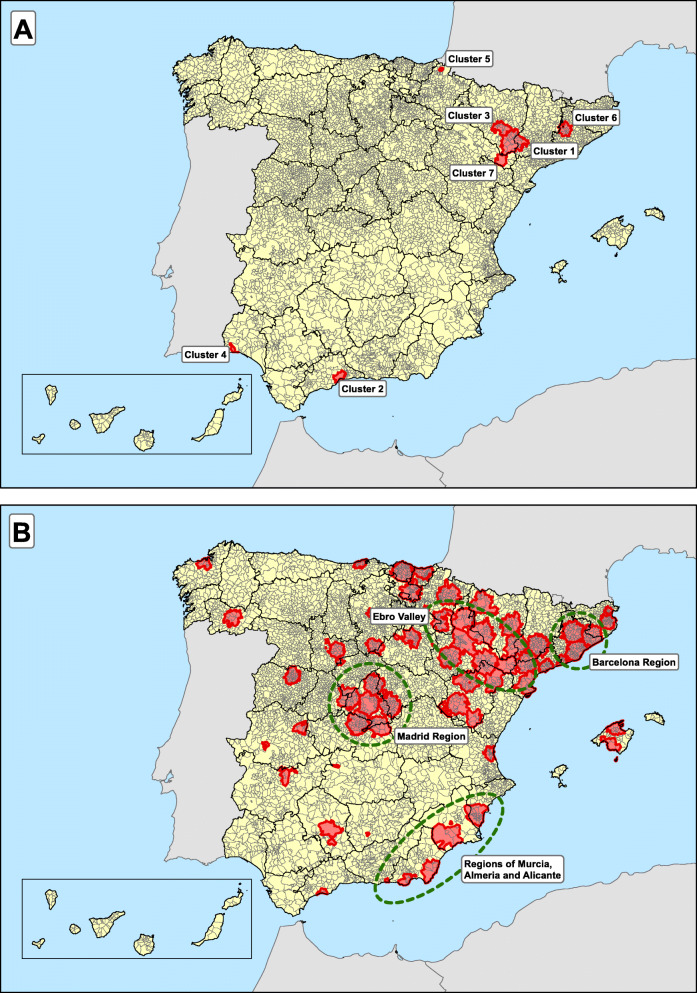
Municipalities included in the top 7 likely space-time clusters of COVID-19 in Spain on June 25th, 2020

A more detailed insight on the study outcomes is discussed below, focusing on two specific days (June 25th and August 1st) as they help describe the spread of the disease in the Spanish territory (Table2 and Map2).

**Table 2:** Emerging space time clusters of COVID-19 at the municipal level in Spain

Cluster	Active cluster period (Days)	***P***	Observed	Expected	RR	# of municipalities	# of municipalities with RR>1
**Active clusters on 25th June 2020**							
1	Jun 23th Jun 25th (3)	<0.001	122	4.26	28.85	28	15
2	Jun 24th Jun 25th (2)	<0.001	62	7.56	8.23	1	1
3	Jun 23th Jun 25th (3)	<0.001	15	0.42	35.44	18	3
4	Jun 24th Jun 25th (2)	<0.001	9	0.36	24.95	1	1
5	Jun 23th Jun 25th (3)	<0.001	4	0.013	300.36	1	1
6	Jun 23th Jun 25th (3)	<0.001	17	2.72	6.26	15	6
7	Jun 23th Jun 25th (3)	0,004	6	0.2	29.61	2	2
**Active clusters on 1st August 2020**							
1	Jul 27th Aug 1st (6)	0.000	2593	79.9	33.64	28	21
2	Jul 27th Aug 1st (6)	0.000	4359	467.4	9.87	86	72
3	Jul 27th Aug 1st (6)	0.000	2928	570.7	5.31	31	30
4	Jul 27th Aug 1st (6)	0.000	574	24.96	23.18	40	31
5	Jul 27th Aug 1st (6)	0.000	760	103.02	7.45	89	41
6	Jul 27th Aug 1st (6)	0.000	725	152.98	4.78	32	25
7	Jul 29th Aug 1st (4)	0.000	56	0.028	2026.62	1	1
8	Jul 27th Aug 1st (6)	0.000	55	0.026	2094.31	1	1
9	Jul 27th Aug 1st (6)	0.000	301	49.55	6.1	4	3
10	Jul 27th Aug 1st (6)	0.000	176	13.73	12.84	73	23
11	Jul 27th Aug 1st (6)	0.000	193	20.15	9.6	7	5
12	Jul 27th Aug 1st (6)	0.000	210	30.13	6.99	24	4
13	Jul 27th Aug 1st (6)	0.000	236	40.04	5.91	58	20
14	Jul 27th Aug 1st (6)	0.000	122	7.78	15.71	17	10
15	Jul 27th Aug 1st (6)	0.000	183	24.72	7.42	7	5
16	Jul 28th Aug 1st (5)	0.000	101	6.94	14.57	28	7
17	Jul 27th Aug 1st (6)	0.000	84	4.2	20.04	25	5
18	Jul 27th Aug 1st (6)	0.000	265	68.92	3.86	34	24
19	Jul 27th Aug 1st (6)	0.000	127	15.53	8.19	2	2
20	Jul 27th Aug 1st (6)	0.000	53	1.26	42.09	9	4
21	Jul 27th Aug 1st (6)	0.000	72	4.71	15.29	25	9
22	Jul 27th Aug 1st (6)	0.000	71	4.6	15.45	29	4
23	Jul 27th Aug 1st (6)	0.000	54	1.98	27.33	8	5
24	Jul 27th Aug 1st (6)	0.000	208	57.8	3.61	62	30
25	Jul 27th Aug 1st (6)	0.000	50	2.37	21.11	26	11
26	Jul 27th Aug 1st (6)	0.000	95	13.69	6.95	21	13
27	Jul 27th Aug 1st (6)	0.000	39	1.3	29.93	39	7
28	Jul 27th Aug 1st (6)	0.000	86	12.44	6.92	27	19
29	Jul 28th Aug 1st (5)	0.000	17	0.039	433.45	1	1
30	Jul 27th Aug 1st (6)	0.000	179	56.76	3.16	56	34
31	Jul 27th Aug 1st (6)	0.000	115	25.62	4.49	30	16
32	Jul 27th Aug 1st (6)	0.000	59	5.84	10.11	8	4
33	Jul 27th Aug 1st (6)	0.000	116	28.83	4.03	20	11
34	Jul 27th Aug 1st (6)	0.000	51	4.87	10.47	29	7
35	Jul 27th Aug 1st (6)	0.000	53	6.41	8.28	47	13
36	Jul 27th Aug 1st (6)	0.000	132	44.53	2.97	55	15
37	Jul 27th Aug 1st (6)	0.000	40	4.21	9.51	3	3
38	Jul 27th Aug 1st (6)	0.000	30	2.2	13.62	16	2
39	Jul 31th Aug 1st (2)	0.000	21	0.8	26.12	1	1
40	Jul 27th Aug 1st (6)	0.000	89	28.98	3.07	59	17
41	Jul 27th Aug 1st (6)	0.000	31	3.75	8.27	53	15
42	Jul 31th Aug 1st (2)	0.000	38	6.59	5.77	39	5
43	Jul 27th Aug 1st (6)	0.000	108	44.71	2.42	39	14
44	Jul 31th Aug 1st (2)	0.000	10	0.16	63.61	1	1
45	Jul 27th Aug 1st (6)	0.000	97	39.17	2.48	25	8
46	Jul 28th Aug 1st (5)	0.000	78	28.65	2.72	1	1
47	Jul 30th Aug 1st (3)	0.000	59	18.77	3.15	14	10
48	Jul 27th Aug 1st (6)	0.000	97	43.82	2.22	45	17
49	Jul 28th Aug 1st (5)	0.000	7	0.1	68.38	1	1
50	Jul 29th Aug 1st (4)	0.000	13	0.92	14.08	10	5
51	Jul 28th Aug 1st (5)	0.000	10	0.43	23.26	9	5
52	Jul 30th Aug 1st (3)	0.000	36	9.45	3.81	9	4
53	Jul 30th Aug 1st (3)	0.000	57	20.7	2.75	8	6
54	Jul 28th Aug 1st (5)	0.000	51	17.61	2.9	50	10
55	Jul 28th Aug 1st (5)	0.000	20	3.15	6.34	20	6
56	Jul 28th Aug 1st (5)	0.000	18	2.54	7.09	6	3
57	Jul 28th Aug 1st (5)	0.000	17	2.37	7.18	16	4
58	Jul 30th Aug 1st (3)	0.000	23	4.88	4.72	54	12
59	Jul 27th Aug 1st (6)	0.001	9	0.53	17.02	1	1
60	Jul 30th Aug 1st (3)	0.001	8	0.38	20.96	1	1
61	Jul 27th Aug 1st (6)	0.004	46	17.99	2.56	20	9
62	Jul 28th Aug 1st (5)	0.004	33	10.61	3.11	13	7

*RR* Relative Risk

### Municipal- level results. June 25th, 2020

The scan analysis for the 25th of June detected seven statistically significant emerging space-time clusters of COVID-19 are described at Table2 and shown in Map2a. The most likely cluster (cluster 1) was located between two administrative regions: Huesca (Aragn) and Lleida (Catalua). It presented a relative risk (RR) of 28.8, with 122 observed cases compared to 4.26 expected, and it was active from June 23rd to the date of analysis. Although most cases were located in 3 municipalities, 15 out of the remaining 25 included municipalities had a RR>1.

Other small clusters are found in the south, cluster number 2 and 4 (Mlaga and Huelva) and cluster 5 in the north of Spain (Navarra), each including a single municipality. They involved 62, 9 and 4 cases, respectively, with a RR of 8.23, 24.95 and 300.36. The start date of the active cluster varies: June 24th (cluster 2 and 4) and June 23rd (cluster 5); while the following analysis date was the same (June 25th).

Cluster 3 contains 18 municipalities, one of them shows the highest individual RR for the day of analysis (478.07). The global cluster RR was 35.44, with only 3 municipalities with a RR>1.

### Municipal-level results. August 1st, 2020

By August 1st, 2020, 62 clusters of COVID-19 were active in Spain, present over the whole territory but two Autonomous Regions and the Autonomous Cities. They are described in Table2 and shown in Map2b. The number of cases ranged from 7 in the smallest aggregation to 4359 in the largest one. Cluster duration had increased and more than half of them had started 5 days before (on June 27th).

An accumulation of clusters is seen in the Ebro Valley. In fact, the most likely cluster (*p*<0.001) was located around the city of Zaragoza. There were 2593 incident cases from July 27th to August 1st, it had a RR of 33.64 and 21 out of the 28 municipalities included had a RR>1. Surrounding it, there were several smaller significant clusters. One of them was the cluster 4, active for the same period, which comprised Lleida city and their close municipalities (574 cases, RR: 23.18). This area had been the most likely cluster when the analysis began.

The Mediterranean coast of Catalua was also affected during this period. Cluster 2 is located in Barcelona and its surroundings. A total of 4359 cases were reported, affecting 86 municipalities (72 with a RR>1) and with a RR of 9.87 for the complete aggregation.

Cluster 3 is located in the Madrid Autonomous Region and is formed by 2928 cases (RR: 5.31). It encompasses Madrid and its metropolitan area (30 of 31 municipalities with RR>1). Around this cluster, there are 4 less-likely ones, at the rural areas of this administrative region. The remaining significant clusters can be consulted at Map2b.

By that day, we can observe that practically most of north-eastern Spain was affected by COVID-19 clusters as well as Madrid and the Mediterranean coast, especially the south-eastern regions of Murcia, Almeria and Alicante.

### Parameter evolution during the study period

**Fig. 2:**
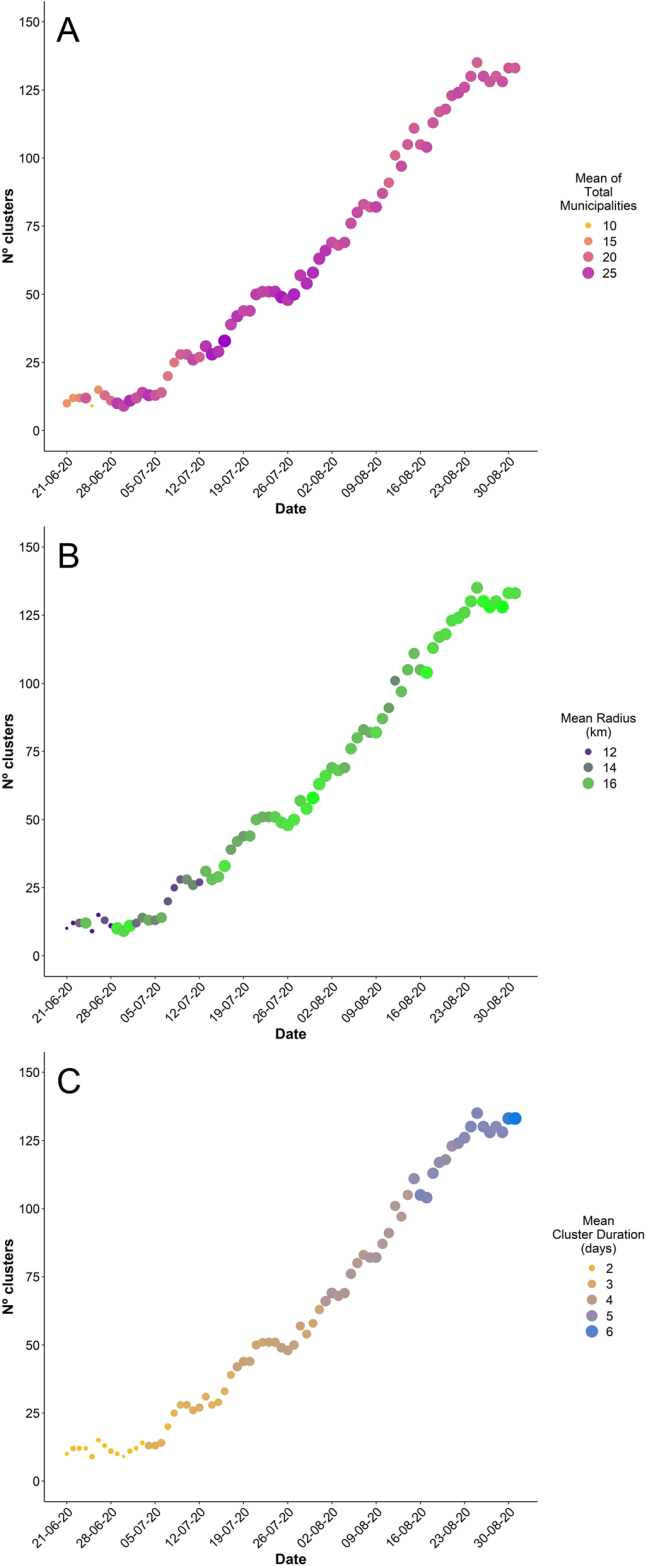
Parameter evolution during the study period. Scatterplot of the distribution for the **a** mean of total municipalities per cluster, **b** mean cluster radio and **c** mean duration for the clusters obtained for each day of the analysis

Cluster radius and number of municipalities per cluster allow us to assess another quantitative dimension of pandemic evolution (Fig.2). Assuming an initial scenario of low transmission during the first 23weeks of our study period, a grouping of small clusters can be observed, with a mean radius of 10 to 14km that includes 10 to 15 municipalities (Fig.2a and b). Around mid-July an increase is noted in both parameters, with a mean radius ranging from 18 to 20km and a daily mean of 30 municipalities per cluster, this value will remain up to August 31st (Fig.2a and b). The third included parameter (Fig.2c), mean time duration of active clusters, presents the same evolution but in a more progressive trend, as a linear increase can be appreciated. During the first days of the study, clusters only were active for a mean of 23days, but as epidemic evolved this duration was progressively increased. By mid-August its mean value almost reached the 7days limit imposed on our model.

## Discussion

On June 21st, State of Alarm and de-escalation measures ended in Spain and only 9 active clusters were detected, compared to 129 of them present by August 31st^.^ Surveillance set up in May was updated on June [5] and a new legal framework to prevent, contain and coordinate public health measures against COVID-19 was developed [23]. Autonomous Regions were responsible for reporting all detected outbreaks to the Ministry of Health. However, it is important to note that the terms outbreak and cluster denote different concepts: whereas cluster refers to a statistical outcome of our space-time analysis, outbreaks in Spain were defined as the aggregation of at least 3 cases with an epidemiological link between them [5].

### Evolution of pandemic and populations affected in Spain during 2020 summer

We have applied STSS to detect emerging outbreaks of COVID-19 in Spain after de-escalation. By June 21th, 9 clusters were active, located in specific regions. They represent some of the very first outbreaks that took place since then. From there, transmission spread geographically to neighboring regions during July, making the total daily clusters rise to 59 by July 31st, mostly in Ebros Valley but a trace can also be observed along the Mediterranean coast and Madrid. In August, when Madrid became the most likely cluster, diffusion in the regions adjacent to the capital can be appreciated, leading to a heterogeneous cluster prevalence by the end of the study period.

The main and first cluster in our analysis, that took place in the cities of Lleida and neighboring municipalities of Huesca, was strongly linked to vulnerable collectives, both meat-processing plants and agricultural industry/seasonal workers [24]. Occupational-related outbreaks were the most commonly observed up to July [6]. They have been widely reported in Europe during the 2020 summer, mainly related to food packaging and processing sectors, factories and manufacturing, office settings and health workers [25]. In addition, COVID-19 spread has been more intense between vulnerable collectives and low-income populations [26].

From mid-July, social and family outbreaks largely outnumbered occupational ones, with 40% out of the total, as transmission began to grow [6] after June 21st and the return of mobility in Spain. This increase during July coincides at the time that cluster number, mean cluster radius and mean locations per cluster reached upper bounds according to parameter limitation. As transmission grew, initial outbreak detection was no longer informative since community transmission was suspected to be occurring, which has implications in surveillance.

### SaTScan method. Strengths and applications for COVID-19 surveillance

By August (https://coviddifusion.isciii.es/SpSumClus/), we observed the appearance of multiple neighboring clusters. Cluster mean radius and number of included municipalities by cluster had reached a maximum, suggesting a probable widespread transmission. STSS could become a valuable tool to respond to an important question in any epidemic outbreak: when and how to determine if a community transmission might be happening. Using prospective STSS, Masrur et al. suggest that a COVID-19 community transmission might had happened in Bangladesh in March [17]. This scenario is of enormous importance for public health authorities and decision-makers, and we believe that spatial aggregation saturation of clusters over an area, once reached maximum established parameters (cluster radius fundamentally), can be understood as a relevant indicator to consider that community level transmission could be happening.

Analysis options and parameters may be adjusted depending on desired surveillance objectives. Cluster diameter may be changed to reach specific surveillance goals: in an outbreak control strategy, a smaller radius can help detect single municipalities whose RR is increasing rapidly. However, once community transmission has been detected, it may be more useful to use a larger diameter to detect an aggravation of the situation at wider regions. Moreover, time range and a minimum of included cases could be set in order to look for aggregation of different sizes. A 5 cases/7days ratio may be a good option to detect family outbreaks in a low-density area, while 300 cases/ 7days could be a useful tool in a decision-making panel.

Figure2 represents, to the best of our knowledge, the first attempt to evaluate timely performance of STSS surveillance under established parameters. By mid-July, a window widening might be necessary to better capture emerging behavior since parameters already show a saturation (i.e., they reached their upper limit for the entire period). Other research teams chose to group clusters by population-at-risk included, with a minimum of 10% of the total population needed, resulting in less but wider clusters [15,17]. Population in Spain is unequally distributed, mainly within the coastal regions and Madrid. If we had chosen this population-at-risk option, emerging clusters affecting single small municipalities would have never been detected at the beginning of the study period.

The use of surveillance records is subject to reporting bias. During low prevalence time periods, data can be expected to arrive daily in complete form. But as epidemic progresses, notification delays become present and data quality might decline, which may complicate a real-time assessment. Usual weekend bias is also accounted for.

## Method limitations

Prospective SSTS is very influenced by the epidemic curve shape. As this disease is distributed in waves, its validity may be compromised by trend changes. For this reason, it seems logical to restart the analysis every time this situation occurs. Furthermore, community transmission can compromise its utility. Once established, cluster detection loses usefulness for showing the emerging distribution of the disease.

Expected cases are calculated from previous incidence of COVID-19. The use of real-time surveillance data is subjected to reporting delays and bias. Since we used a consolidated database, this effect has not been measured and needs further research and experience.

The use of a minimum radius between municipalities can be affected by the territorial distribution in Spain, which is very heterogeneous. The notorious differences in the size of municipal terms between South and North regions could separate more frequently clusters where they are bigger, as Andaluca (see Map1). In addition, population concentration may cause great variations in RR as a higher number of cases is needed to make a cluster significant in most populated areas with respect to less ones. Besides, the high RR of a few close municipalities can reach surrounding villages or towns with a RR<1 or even without a case inside a cluster. Control and prevention measures should take this into account, and be intimately linked to the detection of transmission chains and the epidemiological situation.

Clusters, as STSS output, have to be considered as a statistical result, where there is an absence of epidemiological links or a deep knowledge of the real situation. Its use should be associated with a suitable and well-staffed prevention and control strategy, which allows to provide a true vision of the epidemiological situation.

## Conclusions

STSS surveillance captured the near-real time evolution of COVID-19 cases clusters during the summer 2020 in Spain, following the end of the national lock-down. A total of 100 new cluster (from 9 to 129) were detected during the study period, following observed spatial diffusion for the second wave.

Space time scan statistics provides timely and reliable information for real-time surveillance of COVID-19 emerging clusters and later pandemic evolution. Epidemiological investigation is needed to characterize clusters and guide interventions. Its flexibility allows for a variety of surveillance strategies and populations. Once emerging outbreaks lead to a wider transmission, STSS could become an early warning of community transmission.

STSS surveillance serves as a public health evidence-based decision tool, and will be implemented as part of Spanish routine COVID-19 surveillance. Further research will be conducted to evaluate its performance and utility in decision making for epidemic control.

## Data Availability

The data that support the findings of this study are available at the COVID-19 section of the web page from the Centro Nacional de Epidemiologa, Instituto de Salud Carlos III (Spain): https://cnecovid.isciii.es/covid19/#documentaci%C3%B3n-y-datos

## Abbreviations

PCR: Polymerase chain reaction
PHEIC: Public Health Emergency of International Concern
RENAVE: Spanish National Epidemiological Surveillance Network
RR: Relative risk
SiViES: Spanish Surveillance System electronic platform
STSS: Space-time scan statistic
